# Why Has Personality Psychology Played an Outsized Role in the Credibility Revolution?

**DOI:** 10.5964/ps.6001

**Published:** 2021-08-12

**Authors:** Olivia E. Atherton, Joanne M. Chung, Kelci Harris, Julia M. Rohrer, David M. Condon, Felix Cheung, Simine Vazire, Richard E. Lucas, M. Brent Donnellan, Daniel K. Mroczek, Christopher J. Soto, Stephen Antonoplis, Rodica Ioana Damian, David C. Funder, Sanjay Srivastava, R. Chris Fraley, Hayley Jach, Brent W. Roberts, Luke D. Smillie, Jessie Sun, Jennifer L. Tackett, Sara J. Weston, K. Paige Harden, Katherine S. Corker

**Affiliations:** [1] Department of Medical Social Sciences, Northwestern University, Chicago, IL, USA.; [2] Department of Psychology, University of Toronto, Mississauga, Canada.; [3] Department of Psychology, University of Victoria, Victoria, Canada.; [4] Department of Psychology, Leipzig University, Leipzig, Germany.; [5] Department of Psychology, University of Oregon, Eugene, OR, USA.; [6] Department of Psychology, University of Toronto, Toronto, Canada.; [7] School of Psychological Sciences, University of Melbourne, Melbourne, Australia.; [8] Department of Psychology, Michigan State University, East Lansing, MI, USA.; [9] Department of Psychology, Northwestern University, Evanston, IL, USA.; [10] Department of Psychology, Colby College, Waterville, ME, USA.; [11] Department of Psychology, University of California, Berkeley, CA, USA.; [12] Department of Psychology, University of Houston, Houston, TX, USA.; [13] Department of Psychology, University of California, Riverside, CA, USA.; [14] Department of Psychology, University of Illinois, Champaign, IL, USA.; [15] Hector Research Institute of Education and Sciences and Psychology, University of Tübingen, Tübingen, Germany.; [16] Department of Psychology, University of Pennsylvania, Philadelphia, PA, USA.; [17] Department of Psychology, University of Texas, Austin, TX, USA.; [18] Population Research Center, University of Texas, Austin, TX, USA.; [19] Department of Psychology, Grand Valley State University, Allendale, MI, USA.

**Keywords:** personality psychology, credibility revolution, meta-science, replication crisis

## Abstract

Personality is not the most popular subfield of psychology. But, in one way or another, personality psychologists have played an outsized role in the ongoing “credibility revolution” in psychology. Not only have individual personality psychologists taken on visible roles in the movement, but our field’s practices and norms have now become models for other fields to emulate (or, for those who share Baumeister’s (2016, https://doi.org/10.1016/j.jesp.2016.02.003) skeptical view of the consequences of increasing rigor, a model for what to avoid). In this article we discuss some unique features of our field that may have placed us in an ideal position to be leaders in this movement. We do so from a subjective perspective, describing our impressions and opinions about possible explanations for personality psychology’s disproportionate role in the credibility revolution. We also discuss some ways in which personality psychology remains less-than-optimal, and how we can address these flaws.

Social psychology might think carefully about how much to follow in personality psychology’s footsteps. Our entire field might end up being one of the losers.

— Baumeister (2016, p. 158)

When it comes to its role in the credibility revolution, personality psychology is punching above its weight. To an interested observer watching the unfolding of this movement, it might be difficult to make sense of personality psychology’s outsized role. Some of the most visible advocates for reform have come from personality psychology, despite the fact that we are a very small, and typically not-very-influential, subfield. Moreover, the stereotype of personality research is that it is boring, not especially successful at attracting attention, funding, or respect, and does not share some of the core features of its larger neighbors (e.g., it is rarely experimental like social and cognitive psychology typically are). Some may find it curious that such a minor subfield of psychology has played a disproportionate role in this movement. We do, too. We can understand the feeling, which seems to underlie Baumeister’s warnings in the paper quoted above, that personality psychology is not the subfield you’d expect other subfields to look to for leadership.

The authors of this paper share a curiosity about why personality psychology has had such an outsized role in the credibility revolution. We do not know the answer. However, we believe that sharing our experiences, speculations, and perceptions of our field may help others better understand this period in psychology’s history. In the short-term, we hope to stimulate discussions and alternative explanations for this curious phenomenon. In the long-term, we hope our reflections may provide grist for scholars (e.g., historians, philosophers, and sociologists) studying psychology’s replication crisis and credibility revolution.

This paper should not be considered a definitive or authoritative investigation of the causes of personality psychology’s outsized role. Instead, it should be seen as a subjective account from people on the front lines, reporting their experiences and venturing some guesses about possible explanations. In many places, we chose to present opinions and observations that are not backed up by empirical data in the interest of providing a richer discussion. Any claim presented without a citation or supporting evidence should be treated as speculative and not as fact.

As with most multi-authored opinion pieces, not all authors share all perceptions and opinions articulated here. What we share is an interest in the question of *why* personality psychology may have played a disproportionate role in the credibility revolution and some experiences that speak to possible answers. Our experiences are quite diverse—we have different (sometimes conflicting) intellectual commitments; we are at different stages of our careers and so have witnessed different parts of personality psychology’s history and from different vantage points; we have demographic differences that shape our experiences; and we have different biases, values, and personalities.

We organize this paper into two broad sections: strengths and weaknesses. First, we elaborate on some of the unique strengths of personality psychology and possible explanations as to why we may have taken on the credibility revolution in stride. Specifically, we draw on our history and norms as a field, as well as the methodological and statistical practices that have long been common in personality psychology. Then, in the second section of this paper, we turn to discussing issues that undermine our credibility as a field -- notably, methodological and statistical practices, causal inference, and diversity and generalizability. While our role in the credibility revolution suggests we can set a positive example in some domains, we should not ignore the fact that we have some glaring negative qualities as a field. Thus, this paper captures both optimistic and critical tones about the state of personality psychology’s credibility and our outsized role in the revolution. What factors put personality psychology in a position to play such a role in the credibility revolution? What are some of the pitfalls that personality psychology has not overcome? Where do we most need to improve?

## Strengths of Personality Psychology: What We Do Well

### History and Field Norms

Some of the factors that may have led us to be well-prepared for the credibility revolution stem from events in our history and the cultural norms we have as a field. One episode in our history that left a noticeable mark and arguably set us up for success in the face of the replication crisis, is another crisis that forced us to justify our credibility as a scientific (versus pseudoscientific) field: the person-situation debate. Of course, there are many non-parallels between the person-situation debate and the replication crisis (including the validity of the criticisms leveled against the field), but there are also many striking parallels. For this reason, we suspect that the lessons learned from the person-situation debate played an important role in setting us up to deal with the replication crisis and become involved in the credibility revolution.

The person-situation debate questioned the existence of personality traits, and whether personality versus situational factors mattered more in determining behavior (Lucas & Donnellan, 2009; Mischel, 1968). Thus, the very existence of personality psychology as a field was at stake, at least in the United States. Importantly, personality’s crisis in the 1970s and 1980s in the US was different from the current replication crisis (and many other so-called “crises” that other subfields have faced) in one important way: it threatened the existence of the subfield. The possibility that graduate programs and faculty jobs would disappear, journals would fold, and funding would dry up was very real (e.g., Baumeister, 1999; Swann & Seyle, 2005) - indeed, these developments were under way before things started to turn around in the mid 1980s. Nevertheless, the person-situation debate has many similar features to psychology’s current replication crisis. Most pertinently, many psychologists are now questioning whether everything we’ve been studying is just noise or whether we are studying something real.

For both the person-situation debate and the credibility revolution, these existential questions drew widespread attention and subsequent arguments over how seriously these concerns should be taken (Epstein & O’Brien, 1985; John et al., 2012). Furthermore, discussions about both the person-situation debate and methodological reform were (and are) rather heated. Raw feelings over the person-situation debate have lasted decades (e.g., Lucas & Donnellan, 2009), and critics of the credibility revolution have gone so far as to make public derogatory remarks about scientists interested in methodological reform (e.g., “shameless little bullies,” “replication police,” “methodological terrorists”).

In both cases, the debates over the credibility of the field forced researchers to justify the field’s credibility on scientific grounds. This forced difficult (and rare) conversations about the quality and integrity of research practices and findings, and prompted researchers to formulate competing (and testable) hypotheses regarding the central issues at hand. For example, the person-situation debate led personality scientists to craft ways to empirically test hypotheses about whether personality traits are real (e.g., via cross-situational consistency) and the relative impact of personality traits (vs. situations) on behavior (e.g., Kenrick & Funder, 1988). Likewise for the credibility revolution, critics voiced their dissent using arguments that were subsequently turned into testable hypotheses such as the impact of researcher expertise on the success of replication attempts (Many Labs 4; Klein et al., 2019) and the role of subjective analytic choices on research findings (Silberzahn et al., 2018).

Personality psychologists may have been primed to play an outsized role in the credibility revolution in part because of this sense of familiarity with the revolution we’ve already been through, and because many of the norms and values promoted via the credibility revolution are practices that we have long adopted (likely, at least in part, because of the person-situation debate). The credibility revolution aims to promote a culture where individual scientists and findings are not put on pedestals; where healthy, open critical discourse and incremental research are necessary for scientific progress; where data sharing and replications are valued; and where conclusions about the data are tempered accordingly. Thanks to our experience having to justify our credibility in the wake of the person-situation debate, personality psychologists have engaged with many of these practices before they were cool.

For better and for worse, personality psychologists have mostly failed to gain public attention (and also status/attention within psychology), despite our subject matter being of great interest to the general public, and undeniably important to social and political events. Our inability to translate the popularity of our subject matter into status or influence may be due in part to our struggles establishing our credibility as a field and our place in psychology. This lack of status meant we had fewer opportunities to put individual scientists on pedestals, which has proven to be helpful in the age of the credibility revolution because criticisms and evaluations of personality research were less likely to be seen as personal attacks. Moreover, the fact that personality psychology is a relatively small field, has little money at stake (in terms of grants or external partnerships), isn’t popular within scientific psychology, and largely lacks access to status and attention may have made it so that we generally had less to lose when the replication crisis hit.

Because status is not a major barrier to criticism in our field, this may have also led us to adopt a general culture of strong disagreements within the field, aired quite openly but without a lot of hostility or personal animosity (at least in the last few decades). For example, personality scientists subscribe to different views regarding personality structure and measures (e.g., Big Five, HEXACO, individual trait approaches such as the Q-sort; Anglim & O’Connor, 2019; Wiernik et al., 2020) and have differing opinions about what the most useful levels and units of analysis are (e.g., nomothetic vs. idiographic). Despite these disagreements, personality psychologists cooperate in their scientific pursuits, and critical discourse is carried out through empirical research.

This culture of disagreements and open scientific exchange has arguably led to important advances. Personality psychologists seem to value incremental research, place high value on measurement validity, and have less of a fixation on novelty, thus making us more “boring” (Baumeister, 2016; Funder, 2016). Furthermore, personality psychologists were collecting larger samples, sharing data, reporting null effects, and replicating across studies before these became widely-accepted as being best practices across the rest of psychology. Personality psychologists have also historically valued calibrating conclusions to the data, which is possibly due to intense scrutiny by critics during the person-situation debate.

Post person-situation debate, some may wonder whether personality psychology succeeded in establishing its credibility. From our perspective, it seems as though we succeeded well enough to survive but not well enough to become a popular subfield, or to be well-accepted into the broader field. Time will tell if the credibility revolution and the field of metascience will face the same fate. It is noteworthy that, in the pursuit of establishing credibility, personality psychologists and meta-scientists both developed formal societies. These represent small (but growing) efforts to solidify our respective (and hopefully permanent) places in the field.

### Methods and Statistical Validity

Our long history of “boringness” with respect to methods and statistical validity has proven, in some ways, to pay off, setting us up to meet the replication crisis with confidence. Many of the popular avenues for methodological reform in the age of the credibility revolution are methodological practices that personality psychology has long been obsessed with: 1) psychometrics to establish the existence and validity of traits, 2) using large sample sizes, and 3) a dedication to descriptive and exploratory research.

The field had an early focus on psychometrics, in part because it was necessary. In order to measure traits and develop trait taxonomies, it was critical to take the steps to establish construct validity. Are we measuring what we intend to measure? Cattell (1946) was one of the first to employ factor analysis in the study of traits, a technique that proved formative in the derivation of the Big Five and HEXACO trait models. Additionally, concepts and tools such as convergent and discriminant validity (Loevinger, 1957), the nomological network (Cronbach & Meehl, 1955), and the multi-trait, multi-method matrix (Campbell & Fiske, 1959) have their origins in individual differences research, and these concepts continue to be not only in heavy use in personality today, but have proven to be invaluable tools for robust measurement and rigorous research across many subfields.

Concepts like the nomological network and the multi-trait, multi-method matrix also encourage personality psychologists’ use of multiple methods—including self-report, informant-report, behavioral observation, and biological measures—to triangulate on a claim. For instance, Harari and colleagues (2020) used mobile sensing techniques to examine how personality traits are associated with objective measures of behavior in everyday life. In the area of personality neuroscience, examples abound of researchers triangulating biological measures against self-report or behavioral measures (e.g., Jach et al., 2020). Although the field is sometimes rightly critiqued for an overreliance on self-report questionnaires, the frequent use of multiple methods is notable. Certainly, each method has strengths and weaknesses, but checking the consistency of results across methods have helped the field form a more robust and credible evidence base. These practices serve as a model for other fields seeking to increase their credibility.

Another strength of personality psychology is our track record of using observational data with relatively large sample sizes. A handful of studies have found that research areas and studies that use primarily observational, as opposed to experimental, methods tend to use larger sample sizes (Fraley & Vazire, 2014). In addition, Kossmeier et al. (2019) found a steady increase in sample size over time in two top personality journals. These larger samples may be partly due to more frequent use of statistical techniques that require large samples (e.g., exploratory factor analysis, structural equation modeling), or due to the sometimes lower costs of data collection (e.g., cross-sectional surveys), or due to personality researchers’ tendency to use longitudinal panel studies like the German Socio Economic Panel (GSOEP) or other large existing datasets. Regardless of the reason, larger samples are important for the credibility of research findings because they increase statistical power, thus reducing the incidence of false negatives and increasing the incidence of true positives (which also reduces the false discovery rate, or the proportion of positive findings that are false positives). Additionally, higher powered studies produce more stable effect size estimates (Schönbrodt & Perugini, 2013), which reduces the chances of stumbling on fluke findings and exacerbating publication bias.

Finally, to show the real-world relevance of personality traits, researchers rely on ecologically-valid exploratory and descriptive studies to test the distributions, antecedents, and consequences of traits. This focus on ecologically-valid, descriptive research (which blends confirmatory and exploratory approaches) possibly reduces pressure for personality psychologists to *p*-hack. For instance, some researchers have conducted descriptive studies to document how personality traits vary across nations (e.g., Rentfrow et al., 2013) and across the life course (e.g., Lucas & Donnellan, 2011), and how they predict important life outcomes such as physical health, subjective well-being, and work performance (e.g., Ozer & Benet-Martinez, 2006). Plus, the adoption of broad personality taxonomies, such as the Big Five, makes the file drawer less of a problem and publishing null results much easier (i.e., even if only one of the Big Five traits correlates with the outcome, we can still publish the null effects of the other four traits). For example, researchers may test hypotheses derived from social investment theory regarding how conscientiousness changes when young adults enter the workforce. These confirmatory goals are complemented by an exploration of how the remaining Big Five traits, in addition to conscientiousness, change following joining the workforce. Null results are reasonably common when looking at the antecedents and consequences of five different traits.

Through all of these examples, we can see how our focus on a variety of methodological techniques serves to improve not only reproducibility but also credibility and robustness. A focus on measurement and construct validity provides a solid foundation from which to study personality. A focus on large samples improves statistical inferences. A focus on ecological validity and descriptive research enhances the generalizability and applicability of our research findings and reduces the file drawer problem. These features have led us to be well-prepared to meet the demands of the credibility revolution.

## Weaknesses of Personality Psychology: There’s Still Work to Do

### Methods and Statistical Validity

The many relative strengths of personality as a discipline do not preclude the need for improvement. Although our strengths are heavily concentrated in the domain of methods and statistics, some of our biggest challenges are also in this area, specifically with respect to measurement, design, analytics, and effect size interpretation. For personality psychology to continue to lead in this area, a recognition of the ways that methodological issues affect the practice of personality science is critical.

One notable example is a heavy reliance on verbal reports and especially self-reports to measure core constructs. Although the use of multi-method data is becoming increasingly common, there are a non-trivial number of personality studies that rely on self-reports for both independent and dependent variables. This monomethod design produces artificially inflated effect sizes. Even very small amounts of shared method variance can lead to “replicable confounding” that does not reflect substantively important associations. Moreover, personality psychology is plagued by jingle-jangle fallacies, where sometimes different constructs go by the same name (jingle) and other times, the same constructs go by different names (jangle). Confusion over construct terminology markedly hinders scientific progress in the field (e.g., Leising et al., 2020).

Another particularly salient challenge is related to study design and the reuse of existing data, which presents many opportunities but also has potential pitfalls. Personality psychologists often rely on secondary data analyses of existing, large, longitudinal, and nationally-representative datasets, which means that many key design choices are outside the researcher’s control, such as the selection of measures. Thus, the investigator’s judgment is required to create measures from existing item pools, and the scope of available choices provides ample opportunities for flexibility. When combined with the fact that using large-scale studies often requires choices about which subsamples to include, which waves to analyze, and which covariates to model, there can be a staggering number of decisions to make, thus providing a fertile ground for capitalizing on chance. Moreover, many of these datasets are reused for different purposes by the same or different research teams. Prior knowledge can inform subsequent analyses in ways that facilitate confirmation bias and capitalize on sample-specific characteristics (see Weston et al., 2019, for ways to address these challenges).

Another way the complexity of personality research threatens the accumulation of robust empirical findings is that some longitudinal research designs are difficult to reproduce because of the money and time involved, and thus are rarely replicated. The credibility revolution has shed light on the importance and challenges of replication (Zwaan et al., 2018), primarily because attempts to replicate seemingly simple laboratory experiments failed. Replication is even more complicated when applied to complex personality research. For example, it is exceedingly rare to have longitudinal studies with the exact same measures, data collection intervals, and sampling plans. The apparent lack of commensurability of measurement and design features makes the task of replicating key findings from complex studies all the more difficult. Issues with robustness could go unnoticed due to the practical reality that direct replications of some research designs in personality psychology are hard to conduct.

Moreover, it is not always clear what defines a successful replication. Although debates about how to evaluate whether a replication is successful impact all research areas (Zwaan et al., 2018), this challenge is even greater when studies do not focus on a single, dichotomous test of statistical significance or single effect size estimate. For instance, age-related trajectories in personality traits are thought to be replicable across studies; however, trajectories can be distinguished by general patterns or by their specific shape, age at peak, and more (Graham et al., 2020) and impressionistic evaluations of replicability may gloss over important inconsistencies. Similarly, many critical debates in the field center around support for specific factor analytic or structural equation models, and evaluation of these models typically relies on subjective judgments about model fit and the general similarity of coefficients across studies. In short, many of the analyses that personality psychologists conduct necessarily require judgment and subjectivity to interpret, which means that replicability can be overstated. Collectively, these factors threaten the accumulation of robust empirical findings in personality research.

A related concern is that many complicated statistical models are based on assumptions and data-dependent subjective decisions that are not always transparent to readers and reviewers. And often, it is difficult to pre-register analytic plans involving complex modeling because it is nearly impossible to anticipate all of the problems that could arise while analyzing the data. The fact that researchers cannot easily anticipate all of the choices they will need to make and problems they will encounter introduces flexibility despite their best intentions. Researchers often need to constrain certain parameters to get complex models to converge, and whether such constraints are justifiable is open to debate. These problems are addressable with open data, well-documented code, and thorough transparent reporting. However, many of these practices are not yet widely adopted. It is important to distinguish statistical complexity from rigor.

Finally, beyond the double-edged sword of sophisticated modeling approaches, other methodological features in personality research can also have subtle downsides. For instance, the move away from null hypothesis significance testing toward estimating and evaluating effect sizes is a strength of personality psychology (e.g., Funder & Ozer, 2019). However, there is no consensus on what constitutes a meaningful effect size for personality research. This is due, in part, to the fact that the connection between basic research in personality and application to the real-world is often lacking, and also because personality researchers do not attend to effect sizes as much as they should. It is safe to say that most personality effects are not large. This reality is understandable given that many outcomes of practical importance are multiply determined, so researchers should not expect any single variable to be strongly associated with any outcome. However, this fact creates challenges in interpreting whether small and reliable effects of personality are theoretically and practically important, an issue that personality psychologists will need to grapple with. What distinguishes a small but important effect from one that is negligible?

### Causality and Internal Validity

Related to these issues of measurement, study design, complex analytic approaches, and effect sizes is another issue that personality psychologists historically (and presently) grapple with: causality. Personality psychologists often rely on observational designs, as many research questions do not readily lend themselves to randomized experiments. For example, personality psychologists are often interested in the causal effects of traits on behaviors and outcomes, but we lack the means to randomly assign individuals’ personality.^1^ Additionally, we are interested in determinants of personality (e.g., social class, birth order position, maturation) that cannot be directly manipulated either. This raises doubts about the internal validity of our findings.

Although many other subfields of psychology have a single-minded focus on experiments as the only acceptable means to establish causality (and maybe even as the only way to do “proper” research), this is also problematic because, depending on the object of inquiry, experiments may not be the best way to proceed (Rozin, 2001). Experiments do indeed guarantee internal validity under very weak assumptions, but they do not guarantee broader valid causal inferences because of concerns about external validity.

Instead of trying to emulate a strictly experimental mode of science, it may be more instructive for personality psychologists to pay close attention to other disciplines that rely on observational data. For example, epidemiologists tackle many research questions that cannot be solved through experimentation alone, such as whether or not smoking causes lung cancer (Pearl & Mackenzie, 2018), as do economists, political scientists, and sociologists. These fields seem to share a greater acceptance of the goal of causal inference on the basis of observational data, and a stronger reliance on formalized frameworks such as Directed Acyclic Graphs (e.g., Rohrer, 2018) or the Potential Outcomes Framework (Rosenbaum, 2017). Furthermore, they more frequently make use of difference-in-difference designs, instrumental variables, and other study types that can be classified as natural experiments, which further strengthens causal inferences (e.g., Damian et al., 2021; Dunning, 2012). Likewise, the field of behavioral genetics offers genetically-informative designs, which can strengthen causal inferences considerably (e.g., Briley et al., 2018). Personality psychologists are in an excellent position to learn from these adjacent fields and incorporate causal inference as a serious scientific endeavor rather than something that should be hidden behind weaselly language (Grosz et al., 2020).

Nevertheless, taking causal inference seriously will require us to overhaul current practices. For example, while confounding variables are almost universally identified as an important problem, little awareness seems to exist about how to properly select control variables, or how sampling affects causal inference (Rohrer, 2018). Personality psychologists’ enthusiasm for longitudinal data may prove to be a unique strength because such data can improve causal inferences if employed properly (see VanderWeele et al., 2016). However, some overhauling of statistical models may be required as well. While certain classes of models are mainly used for descriptive purposes (such as visualizing trajectories via growth curves), cross-lagged panel models (CLPM) and their modifications (such as the random intercept CLPM; Hamaker et al., 2015) have often been taken as tools to establish causality, despite the fact that they require additional strong assumptions (Usami et al., 2019).

There is no consensus on whether and how to draw causal inferences from observational data, and causal inference generally poses challenges that should not be understated. However, if personality psychologists are willing to accept this challenge and to catch up on developments from other disciplines, we may be able to contribute to yet another “credibility revolution” (such as the one in economics; Angrist & Pischke, 2010), introducing proper causal inference to observational research in psychology.

### Diversity and Generalizability

Even if we were to address the methodological and statistical issues discussed above, the field of personality psychology would continue to suffer from another serious threat to our credibility: the homogeneity of perspectives represented in our field. Diverse perspectives are vital to good science for a number of reasons. First, diversity in who is represented contributes to diversity in the field’s substantive foci; *who* is doing the research impacts *what* research is being conducted (Roberts et al., 2020). A more diverse group of scholars will likely study a broader range of research questions, use a more complete set of tools, and include a more diverse pool of participants. For a field that prizes methodological triangulation, as personality psychology does, a lack of diversity is a serious threat to the comprehensiveness of our research programs. Second, diversity is critical for a robust, self-critical, and self-correcting scientific community (Longino, 1990). Researchers bring their background experiences and assumptions to their own work and to their critical appraisal of others’ work. If personality research is only read and critiqued by a homogenous group of researchers who share similar biases and blind spots, we are less likely to identify and correct flaws in our work. A serious commitment to self-correction, and therefore to credibility, requires a commitment to creating an environment where a diverse range of scholars are included and valued. Personality psychology is failing at this important task.

One major threat to diversity is that the field has often been unwelcoming to women and underrepresented minorities (URMs) - groups whose percentages in our personality undergraduate programs, graduate programs, faculty positions, and professional societies are lower than their percentages in the general population and whose voices have historically not been given the opportunity to be heard. It is our sense that personality psychology contains a highly disproportionate number of researchers who identify as men and White. Specific demographic data are not collected by some of our major societies, such as the Association for Research in Personality (ARP) and the European Association of Personality Psychology (EAPP), suggesting that the demographics of our members either have been considered irrelevant and unnecessary, or have not been considered at all. The lack of data also makes it difficult to track whether efforts made towards equity and inclusion are working. This situation is both a symptom of personality psychology’s past and a major liability to its future. The most overt examples of hostility towards women and underrepresented minorities stem from norms of professional behavior that are common to psychology more broadly, which allow harassment and discriminatory behavior to perpetuate in many professional contexts, including at scientific meetings, in hiring and promotion practices, and with respect to issues affecting scientific prestige.

Furthermore, personality science has historically centered the experiences of White men, extending back to the origins of psychological assessment. Prior to World War II, the most widely-used personality assessments (e.g., Woodsworth’s 1917 Psychoneurotic Inventory and House’s 1927 Mental Hygiene Inventory) contributed to substantial selection biases in military and workplace screenings (Gibby & Zickar, 2008), all while early intelligence tests were weaponized in favor of blatantly racist, discriminatory policies (e.g., Buck v. Bell, 1927). Since then, the field has adopted more inclusive methods for assessing “normative” personality and a broader acknowledgement of the probabilistic relationship between traits and behavioral outcomes. Yet, group differences in psychological traits are still sometimes reported with inadequate or inappropriate interpretations (e.g., J. P. Rushton’s work), raising the possibility for substantial harm by promulgating stereotypes, not only to members of the described groups but also to members of the research community who find such claims offensive.

Relatedly, another problem for generalizable inferences is methodological complacency. For instance, the Big Five measurement model has, for good reasons, been readily embraced by the field, so much so that variants of the Big Five and Big Six frameworks are now used almost exclusively. This persists despite evidence that fails to support the universality of these dimensions (De Raad et al., 2010), much less their true cross-cultural relevance (Laajaj et al., 2019). Although consensus can be a strength by minimizing the degree to which competing camps dig their heels in, it may also be a cause of methodological homogeneity (cf. Thalmayer et al., 2020).

Another double-edged sword is the use of panel study data. These datasets allow researchers to save time and money, and give them access to large nationally representative samples, but personality psychologists have overwhelmingly focused on analyzing panel data from Western countries (e.g., the German Socio-Economic Panel [SOEP], Midlife in the US, and Dutch Longitudinal Internet studies for the Social Sciences [LISS] datasets), despite the existence of several non-Western panel datasets (e.g., Midlife in Japan Study, the Indonesian Family Life Study, the Chinese Family Panel Study, the Korean Longitudinal Survey of Women and Families, and the sister studies to the Health and Retirement Study collected across the globe).

Moreover, as of October 2020, a review of the 50 most cited empirical papers that list personality as a keyword indicates that all 50 papers were authored by people with institutional affiliations in the United States, Canada, Germany, the UK, and New Zealand, and only three papers included samples outside of these regions (see Supplementary Materials). Interestingly, only one paper title mentions the sociodemographic characteristics of the people being sampled, and the other titles generally refer to psychological constructs rather than people, consistent with the idea that, “One consequence of a USA-centric sampling bias in psychology may be biased assumptions of (White) people from the United States as especially reflective of humankind” (Cheon et al., 2020, p. 1).

A step in the right direction would be to acknowledge the self-perpetuating features of these concerns. An attainable first move would be for our professional organizations to document member demographics (e.g., gender, racial and other identities, nationality, etc.). This would provide a clearer sense of the diversity of people who are currently in the field, as well as a benchmark to assess our progress in the future. This is, however, a very basic first step. It is also important to build on this initial step by making this information accessible and transparent to members of the field, so that in our professional society and departmental contexts, we can make informed decisions about what steps need to be taken to diversify. In other words, collecting the data is not enough; it must be accessible, transparent, and turned into action to have an impact.

Another, perhaps more difficult, endeavor is to reflect on the culture that we may be perpetuating in our classes, laboratories, departments, and research output. For example, in our teaching and knowledge mobilization, we could take care to avoid characterizing certain traits as wholly adaptive (or maladaptive) without communicating that social and contextual factors are necessary for understanding what is considered “adaptive” or not (e.g., delay of gratification is not “adaptive” and “desirable” in all social and cultural contexts; Watts et al., 2018). Likewise, we could emphasise the fact that personality traits are not “fixed”. People change in important ways, and these malleable individual differences are critical for understanding social issues, as has been shown in the behavioral genetic literature at the intersection of genetic risk, education, and social mobility (e.g., Herd et al., 2019).

Furthermore, we could consider the extent to which we can improve the spaces we inhabit as personality psychologists to make them more welcoming towards people who are underrepresented in the field. One necessary endeavor is to increase URM scholarship in personality psychology and to amplify the voices of URM groups that have historically not been given the opportunity to provide input. This could be encouraged by including URM students in our research activities at the undergraduate level by hiring them as paid research assistants or making use of work-study programs. We can also invite URM scholars to join our editorial boards, present at our conferences, and be our collaborators. In doing so, we can work toward lessening the divide between spaces that are occupied by people who are oriented towards what we might consider “mainstream” personality psychology, and topics that are often investigated by URM scholars (e.g., research on identity may be seen as more central to personality psychology than is ethnic identity research). It is also important that we not only make concerted efforts to invite URM scholars to spaces that majority-group scholars dominate, but to also connect with URM scholars in their spaces (e.g., by attending [currently virtual] conferences such as the American Arab Middle Eastern and North African Psychological Association’s) to better understand what URM scholars find important in relation to personality research and what they need to feel supported in academia. We can continue virtual programming for societies, like ARP and EAPP, to facilitate more international participation and reduce resource barriers that members of underrepresented groups face in attending in-person conventions. We can educate ourselves with the myriad of anti-racism resources available on the Internet and participate in trainings offered by our institutions and within the online community (i.e., Academics for Black Survival and Wellness^2^). Until we fix the field’s diversity problem, personality psychology cannot reach its full potential as a credible, robust science.

## Conclusion

This is our perspective on how personality psychology may have been well-suited to play an outsized role in the credibility revolution, as well as the ways in which our field is in need of improvement. We might be wrong; there are many parts of our perspective that have caveats, “with notable exceptions”, biases, and blind spots. After all, the authors of this piece often had differing perspectives, which we have attempted to incorporate into this piece as much as possible. And, we acknowledge that the factors that set us up for success with the credibility revolution may not be the same factors that will set other fields up for success. There are, of course, individual differences in what works and what doesn’t.

Some may wonder: what is the cost of increasing rigor? It has been noted that,

“Unquestionably the study of personality is far more rigorous today than it was half a century ago. Yet back then personality psychology captured the interest of people in many different fields, influencing anthropology, literary criticism, philosophy, plus lots of other subfields in psychology. The gain in rigor was accomplished by a loss in interest value… its influence on thinkers in other fields is far less, and indeed it has failed to capture the imagination of the intellectual community”

(Baumeister, 2016).

We may indeed be more boring, but from our perspective, what we gain from rigor, in terms of scientific truth, far outweighs that cost. The larger movement towards increasing scientific credibility reaches well beyond social and personality psychology, and even beyond psychology more broadly, to fields like biomedicine, physics, and ecology. If psychologists eschew rigor and fail to embrace transparency, the entire enterprise of psychological research may decline in its influence altogether, even to the point of irrelevance. We could end up being interesting but ultimately untrustworthy, re-invigorating early conceptions of psychology as a pseudo-scientific, rather than scientific, endeavor. To address this, psychologists must challenge themselves and each other to do better, to capitalize on our respective strengths and to acknowledge where we need to make change. We have attempted to do that here for our subfield, and we believe that with the collective efforts of all subfields, we can build a brighter and more credible future.

## Supplementary Material

SOM excel

1

## Relevance Statement

In the future, philosophers of science, historians, and researchers alike may want to understand what it was like “living” through the credibility revolution in psychology and beyond. We provide our subjective view as personality psychologists.

## Key Insights

- An account of personality psychology’s outsized role in the credibility revolution.
- Strengths include historical events, field norms, and methodological/statistical approaches.
- Weaknesses include methodological/statistical approaches, establishing causality, and diversity/generalizability.
- This is our subjective perspective.
- We might be wrong.

## Footnotes

1) Although no random assignment of personality is currently possible, other designs can approximate such manipulations. For example, individuals may be randomly assigned to express higher or lower levels of specific traits (Jacques-Hamilton et al., 2019).

2) See www.academics4blacklives.com
