# Supplementary material for: Why Has Personality Psychology Played an Outsized Role in the Credibility Revolution?
Source: Personal Sci. Author manuscript; Available in PMC 2022 Apr 14. (PMC9008744; doi:10.5964/ps.6001)
Supplement: 1 [file NIHMS1787713-supplement-1.pdf]

Supplementary Materials

For this article the following Supplementary Materials are available via the PsychArchives repository (for access see Index of Supplementary Materials below):

- Open Peer-Review.
- Analysis of 50 most-cited empirical papers using personality as keyword.

Index of Supplementary Materials

Personality Science. (Ed.). (2021). *Supplementary materials to: Why has personality psychology played an outsized role in the credibility revolution?* [Open peer-review]. PsychOpen GOLD. https://doi.org/10.23668/psycharchives.5037

Atherton, O. E., Chung, J. M., Harris, K., Rohrer, J. M., Condon, D. M., Cheung, F., Vazire, S., Lucas, R. E., Donnellan, M. B., Mroczek, D. K., Soto, C. J., Antonoplis, S., Damian, R. I., Funder, D. C., Srivastava, S., Fraley, R. C., Jach, H., Roberts, B. W., Smillie, L. D., . . . Corker, K. S. (2021). *Supplementary materials to: Why has personality psychology played an outsized role in the*

PsychOpen GOLD

credibility revolution? [Additional information]. PsychOpen GOLD. https://doi.org/10.23668/psycharchives.5038
